# Forensic Medicine and Toxicology

**Published:** 1894-03

**Authors:** 


*Forensic Medicine and Toxicology.* By J. DIXON MANN, M.D. Pp. 639. London: CHARLES GRIFFIN & COMPANY, LIMITED. 1893.

This work is an addition to the many useful works forming the "Medical Series" being issued by the Messrs. Griffin for practitioners and students, and is produced by them in a pleasant type and on good paper.

We presume Dr. Mann's Lectures are the original basis of this work, as it partakes so much of the very necessary compression needed by such a subject for such a purpose. To treat the subject of Medical Jurisprudence fully in all its different branches and ramifications, in a philosophical and thorough manner is a gigantic task, and Dr. Mann's book does not at all pretend to be such a work. It is, however, a careful and conscientious work, which seems to us to have gathered up into each section an accurate summary of the chief facts relating to that particular section. In many ways the work offers a handy book of reference for the general practitioner, although it would hardly take the place of the fuller and larger works on the subject in getting up any difficult case. Its chief use will be for the student, to whom it should prove a most useful book for reading for an examination. Good use has been made in it of English and foreign literature, and all the most recent facts have been collected and placed at the disposal of the student.

